# A glycogen derived from sea urchin-*Strongylocentyotus internedius* shifts macrophages to the M1 phenotype and enhances the anti-pancreatic cancer activity of gemcitabine

**DOI:** 10.3389/fphar.2025.1600349

**Published:** 2025-07-25

**Authors:** Zhenzhen Deng, Haoyu Yu, Ning Wu, Qingchi Wang, Jing Wang, Yang Yue, Lihua Geng, Quanbin Zhang

**Affiliations:** ^1^ CAS and Shandong Province Key Laboratory of Experimental Marine Biology, Center for Ocean Mega-Science, Institute of Oceanology, Chinese Academy of Sciences, Qingdao, China; ^2^ Lab for Marine Biology and Biotechnology, Qingdao National Lab for Marine Sci. & Tech, Qingdao, China; ^3^ University of Chinese Academy of Sciences, Beijing, China; ^4^ Laboratory for Marine drugs and biological products, Pilot National Laboratory for Marine Science and Technology (Qingdao), Qingdao, China; ^5^ National Glycoengineering Research Center, Shandong University, Qingdao, Shandong, China

**Keywords:** glycogen, tumor microenvironment, JAK, STAT signaling pathway, gemcitabine, macrophages

## Abstract

One of the biggest obstacles to treating pancreatic ductal adenocarcinoma (PDAC) is chemotherapy resistance. Macrophages are an essential element of the innate immune system and are distributed in almost every tissue in the body. Among them, macrophages infiltrating into the tumor microenvironment negatively regulate tumor immunity and participate in the generation, invasion, migration and drug resistance of PDAC. In prior study, we isolated a polysaccharide from sea urchin-*Strongylocentyotus internedius*, which was identified as a high molecular weight, highly branched glycogen (MSGA). In this study, we found that MSGA increased the expression of iNOS, IL-6, TNFα, IL-12 and triggered macrophage differentiation to the CD86^+^ M1 phenotype. MSGA-induced M1 macrophages decreased the cell viabilities and induced apoptosis of PDAC cells. When combined with gemcitabine (GEM), MSGA significantly enhanced the pro-apoptotic activity of GEM. Mechanistically, MSGA transformed macrophages to the M1 phenotype through the stimulation of the JAK1/3-STAT1 signaling pathway and the suppression of STAT3 activity. Overall, our research showed that MSGA has profound potential for tumor immunotherapy. And as an “immune stimulator”, MSGA could assist GEM in the treatment of PDAC.

## 1 Introduction

Pancreatic ductal adenocarcinoma (PDAC) is a highly malignant tumor and one of the most aggressive tumors. PDAC is the third most common cause of mortality in both men and women, killing approximately 50,000 people each year (Siegel et al., 2022). The overall 5-year survival rate is less than 10%, and late clinical presentation, early metastasis and recurrence are mainly responsible for poor prognosis (Ghaneh et al., 2008; Pascual et al., 2004). Radical surgery, radiotherapy and chemotherapy are still the main treatment methods of PDAC. Among them, chemotherapy has been the biggest contributor to improving the treatment of PDCA. Gemcitabine (GEM)-based chemotherapy regimens such as gemcitabine combined with nab-paclitaxel, GEM combined with capecitabine, GEM combined with erotinib, etc., play an important role in the first-line treatment of metastatic pancreatic cancer. However, the overall response rate to GEM therapy for PDAC is less than 20%, resulting in poor prognosis (Catenacci et al., 2015). Although it has been demonstrated that modified FOLFIRINOX regimens (oxaliplatin, leucovorin, irinotecan, and 5-fluorouracil) are more effective than GEM for the treatment of PDAC, their greater toxicity prevents them from being widely used (Conroy et al., 2018). Therefore, GEM monotherapy or combination therapy remains the cornerstone of current chemotherapy for PDAC. Overcoming chemotherapy resistance or enhancing chemosensitivity of GEM remains a major challenge for PDAC therapy.

Macrophages, which account for about 50% of the total tumor mass, play a negative regulatory role in tumor (Takaya et al., 1998; Liu et al., 2007; Liu et al., 2008). Macrophages that infiltrated the tumor microenvironment, also known as tumor-associated macrophages (TAMs), usually show the M2 phenotype. TAMs secret cytokines and chemokines to recruit Th2 immune cells and induce immunosuppression in tumor site (Kakutani et al., 2012a). TAMs promote the growth, invasion and migration of cancer cells and induce chemotherapy resistance through matrix remodeling (Kakutani et al., 2012b). Surprisingly, macrophages are plastic and could be programmed into a classically activated M1 phenotype, which exhibit tumor rejection naturally. The density of M1-type macrophages in tumor stroma is an independent prognostic indicator of PDAC and is positively correlated with long-term survival and favorable prognosis (Wang et al., 2021; Cassetta and Pollard, 2018). M1 macrophages showed considerable potential for chemotherapy sensitization. It has been reported that the infiltration of M1-type macrophages increased the response of cancer cells to etoposide, 5-fluorouracil, doxorubicin, cisplatin and other drugs, and enhanced the anti-tumor activity of chemotherapy drugs by reducing drug metabolic and molecular competition (Cassetta and Pollard, 2018; Laskin et al., 2011; Chen and Zhang, 2017; Mantovani et al., 2010). These studies suggested that elevating the number of M1-macrophages in the tumor site may reverse the immunosuppressed tumor microenvironment and improve chemotherapy resistance.

Glycogens is stored in large quantities in animals and microorganisms, and its primary function is generally considered to be energy store. Previous researches have revealed that glycogen also has immune-stimulating and anti-cancer properties (Laskin et al., 2011; Chen and Zhang, 2017). According to previous reports, glycogen, as an immunostimulatory polysaccharide can act on the immune system, triggering several cellular/molecular events, leading to immune system activation of anticancer response (Mantovani et al., 2010; Vinogradov et al., 2014; Huang et al., 2021). Monocytes, macrophages and T cells are the main target molecules responsible for these reactions. The molecular mechanisms include enhancing the phosphorylation levels of Erk, NFκB and MAPK. However, its molecular mechanism is still lacking. Previously, we separated a glycogen (MSGA) from Strongylocentyotus internedius (Li et al., 2022a). Initial research revealed that MSGA had immune regulatory activity. However, the regulation of MSGA on phenotypic differentiation of macrophages and its anti-PDAC activity remain unknown.

In this study, we studied the role of MSGA in phenotypic differentiation of macrophages and its anti-PDAC activity when used as an immunostimulator in conjunction with the chemotherapy drug gemcitabine.

## 2 Materials and methods

### 2.1 Preparation of MSGA

MSGA was prepared according to the method of Wang et al. (Liu et al., 2008). All animals received care in accordance with the recommendations of the National Institutes of Health Guide for Care and Use of Laboratory Animals, and the experiment scheme was approved by the Committee on the Ethics of Animal Experiments of Institute of Oceanology, Chinese Academy of Sciences (CTEC-2022 (02–01)). Defatted sea urchin egg powder was extracted in hot water (90°C), treated with papain and alkaline phosphatase, and then subjected to ethanol precipitation. The precipitate was further purified using a DEAE Fast Flow anion-exchange column (5.0 cm × 50 cm) (GE Healthcare, Connecticut, United States) and a Sephadex G200 gel filtration column (2.6 cm × 100 cm) (GE Healthcare, Connecticut, United States) to obtain MSGA.

### 2.2 Cell culture

Raw264.7 and pancreatic ductal carcinoma cancer (PDAC) cell lines (PANC-1 cells, MIA-PaCa-2, ASPC-1 and SW1990 cells) were purchased from the Cell Bank of Chinese Academy of Sciences (Shanghai, China). The Raw264.7, ASPC-1 and SW1990 cells were maintained in 1,640 medium (Hyclone, United States) supplemented with 100 U/mL penicillin, 100 μg/mL streptomycin and 10% foetal bovine serum (FBS) (Gibco, United States). PANC-1 and MIA-PaCa-2 cells were grown in DMEM high glucose medium contained 100 units/mL penicillin, 100 μg/mL streptomycin and 10% FBS. Cells were cultured at 37 °C in a 5% CO2 incubator.

### 2.3 Cell differentiation

Different inducible factors were used to induce macrophages to different phenotypes. M1 macrophages were gotten by incubating Raw264.7 cells with 20 ng/mL IFN-γ (Peprotech, United States) and 100 ng/mL LPS (Yuanye, China) for 24 h Raw264.7 cells were cultured with 20 ng/mL IL-4 (Peprotech, United States) and 20 ng/mL IL-13 (Peprotech, United States) to obtain M2 macrophages.

We induced mouse bone marrow macrophages (BMDMs) by referring to the Su’s Methods (Su et al., 2019). C57BL/6J male mice (6–8 weeks) had their femur and tibia harvested to obtain bone marrow cells. After being cultured for 5 days in RPMI 1640 medium including 20 ng/mL M-CSF, monocytes were converted into BMDMs. The induction of BMDMs with different phenotypes was referred to the induction method of Raw264.7 cells.

### 2.4 Cell identification

Anti-CD86-FITC (ebioscience, 11–0862–82) and anti-CD206-PE (ebioscience, 12–2061–82) were labeled to macrophages. The phenotype of macrophages was identified using flow cytometry (BD, United States). Further, Macrophages with different phenotypes were detected by immunofluorescence (IF). Macrophages were labeled anti-CD86 antibody (CST, 91,882) and anti-CD206 antibody (CST, 24,595), respectively. Next, macrophages were incubated with secondary antibody coupled with FITC or Alex fluor 594 at 25°C for 1 h. The expressions of CD86 and CD206 were observed by confocal laser microscope (Leica, Germany).

### 2.5 Cell viability assay

MTT assay was employed to determine the cell viability. A density of 1 × 10^7^ of RAW264.7 cells and PDAC cells were seeded in a 96-well plate overnight and then cultured in various CMs or MSGA (0, 100 and 400 μg/mL) for 36 h. Each well received a 10 μL aliquot of MTT solution (5 mg/mL), which was then incubated for an additional 4 h. The formazan was dissolved with 150 μL DMSO per well, and the absorbance at 570 nm was determined by using a microplate reader (Tecan, Switzerland).

### 2.6 Cytokine assay and NO assay

The M1-specific cytokines (TNFα, IL-6) in cell supernatant were determined by mouse TNFα/IL-6 enzyme-linked immunosorbent assay kit (BOSTER, China), and the content of NO was determined by NO detection kit (Beyotime, China). The above determinations were tested according to the manufacturer’s instructions.

### 2.7 Scanning electron microscopy (SEM)

Raw264.7 cells were immobilized in 2.5% glutaraldehyde, and the cell morphology was observed by SEM (S-3400N, Japan) after dehydration and drying.

### 2.8 Quantitative PCR assay

Total RNA was extracted by Universial RNA-Extration Kit (Vazyme, China) according to the manufacturer’s instructions. RT SuperMix (Vazyme, China) was used to synthesize first-strand cDNA. The quantitative reaction were conducted by QuantStudio 6 Flex (Bio-Rad, United States). The sequences of the PCR primers were shown in Table 1. Based on internal reference GAPDH, ddCt method was used to calculate the transcription levels of each protein, normalized and calculated the relative quantification.

**TABLE 1:** Primers sequence of cytokines.

Cytokines	Forward sequence (5′-3′)	Reverse sequence (5′-3′)
TGF-β	CTA​AGG​CTC​GCC​AGT​CCC​C	TGCGTTGTTGCGGTCCAC
IL-10	GCA​TGG​CCC​AGA​AAT​CAA​GG	GAG​AAA​TCG​ATG​ACA​GCG​CC
CD206	CAT​GAG​GCT​TCT​CCT​GCT​TCT	TTG​CCG​TCT​GAA​CTG​AGA​TGG
ArginaseⅠ	TTG​GGT​GGA​TGC​TCA​CAC​TG	TTGCCCATGCAGATTCCC
TNFα	ACC​CAC​GGC​TCC​ACC​CTC​TC	CCC​TCT​GGG​GGC​CGA​TCA​CT
iNOS	GGA​ATC​TTG​GAG​CGA​GTT​GT	GCA​GCC​TCT​TGT​CTT​TGA​CC
IL12p40	ATG​GAG​TCA​TAG​GCT​CTG​GAA​A	CCG​GAG​TAA​TTT​GGT​GCT​TCA​C
CD86	TCA​ATG​GGA​CTG​CAT​ATC​TGC​C	GCC​AAA​ATA​CTA​CCA​GCT​CAC​T
GAPDH	AGA​GGG​AAA​TCG​TGC​GTG​AC	CAA​TAG​TGA​TGA​CCT​GGC​CGT

### 2.9 Transwell co-culture system

To mimic the coexistence of macrophages and cancer cells in a tumor microenvironment, a transwell system was developed. In the bottom compartment, 0.6 mL of Raw264.7 cells with various phenotypes were resuspended. And PDAC cells (7 × 10^3^ cells/well) were seeded in the upper chamber. This co-culture system was cultured at 37°C for 36 h. The PDAC cells were washed three times with PBS, fixed with paraformaldehyde, stained with crystal violet and then imaged.

### 2.10 Preparation of conditioned mediums (CMs)

Different phenotypes of macrophages and MSGA-treated macrophages were cultivated in serum free medium for 24 h. The supernatant was prepared as the conditioned medium (CMs).

### 2.11 Annexin V-FITC/Propidium Iodide (PI) apoptosis detection

Cell apoptosis was detected by Annexin V-FITC/Propidium Iodide Apoptosis Detection Kit (Vazyme, China) according to the instructions. Specifically, cells were incubated in Annexin V-FITC and PI staining solutions for 10 min. The cells were evaluated using flow cytometry (BD, United States).

### 2.12 Western blot

Cell proteins were extracted to perform Western blot. RIPA lysis buffer with PMSF and a phosphatase inhibitor was used to lyse the cells. SDS-PAGE was used to separate the proteins, then they were moved to a PVDF membrane. The membrane was incubated with the primary antibody at 4 °C overnight after being blocked with a blocking solution, then the second antibody was applied at room temperature for 1 h. Finally, ECL reagent was used for determination. JAK1 (Affinity, AF5012), p-JAK1 (Affinity, AF 2012), STAT1 (Affinity, AF6300), p-STAT1 (Affinity, AF3300), JAK3 (Affinity, BF0256), p-JAK3 (Affinity, AF8160), STAT3 (Affinity, AF6294) and p-STAT3 (Affinity, AF3293) were measured.

### 2.13 Statistical analyses

The data are presented as the means ± SD (n ≥ 3). The data was analyzed by using GraphPad Prism software (version 6.02, United States). T-test and One-way ANOVA with Tukey’s post-hoc test were used for the statistical analyses. Statistical significance was established for *p < 0.05.

## 3 Results

### 3.1 MSGA polarizes macrophages to CD86^+^ M1 phenotype

**FIGURE 1:**
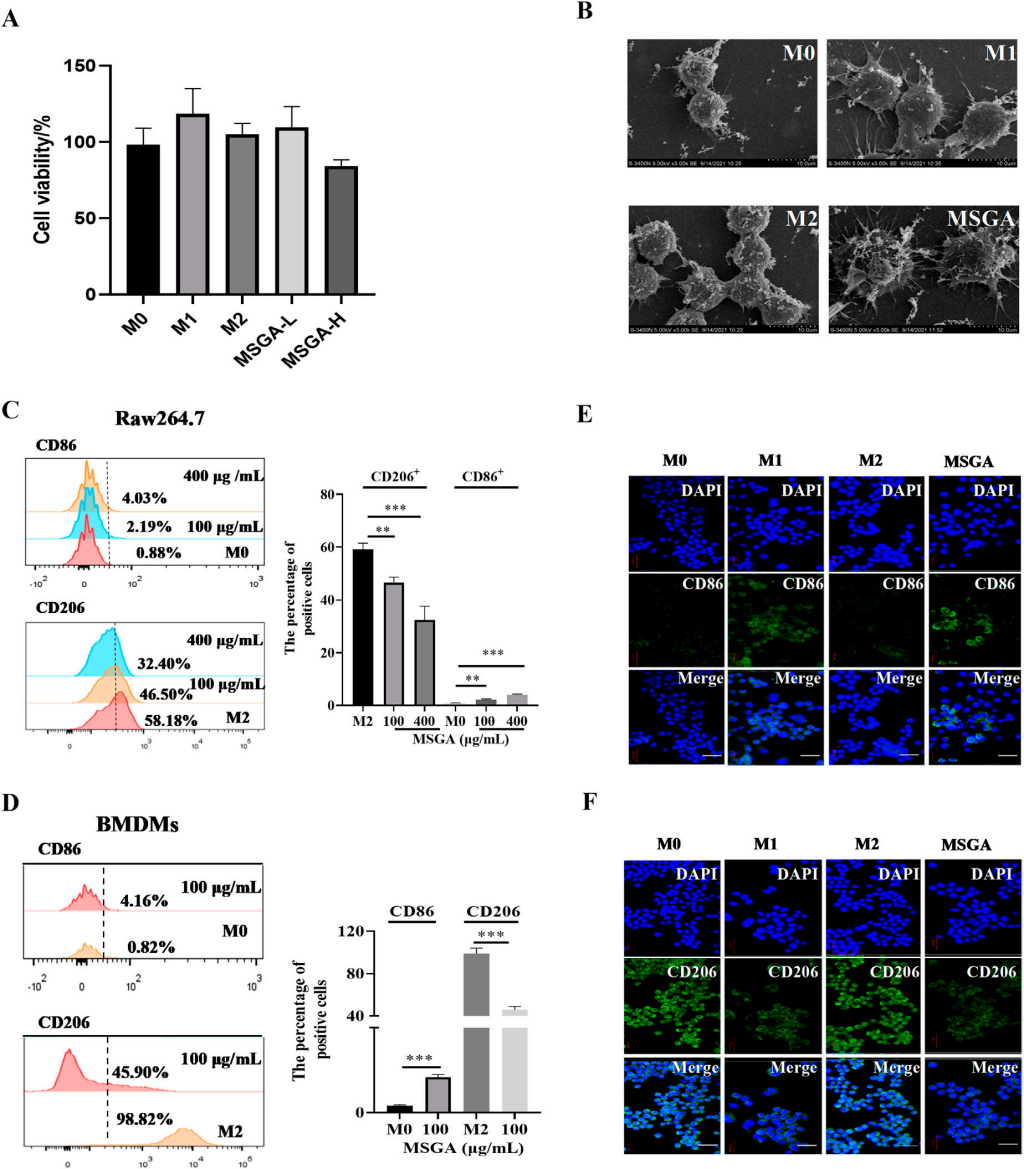
MSGA polarizes macrophages to CD86^+^ M1 phenotype. **(A)** Cell viability of Raw264.7 cells after the cells were treated with MSGA for 36 h, n = 6. **(B)** The morphology of Raw264.7 were observed and collected by scanning electron microscopy (SEM) after treated with MSGA, n = 3. Macrophages were labeled with CD86 and CD206, and flow cytometry and immunofluorescence (IF) were used to identify the phenotypic transformation of macrophage. The percentages of CD86^+^ or CD206^+^ Raw264.7 cells **(C)** or BMDMs **(D)**, n = 3. Representative images captured by laser confocal after immunofluorescence labeling of macrophages with CD86 **(E)** and CD206 **(F)** (image scale, 20 μm), n = 3. MSGA-L (100 μg/mL); MSGA-H (400 μg/mL). Data were expressed as means ± SD. T-test and One-way ANOVA with Tukey’s post-hoc test were used for the statistical analyses. Compared with M0 macrophages, statistically significant differences are indicated. *p < 0.05, **p < 0.01 and ***p < 0.001.

The effect of MDSCs on macrophage viability was investigated first. MSGA had a minor but non-significant impact on the cell viability of Raw264.7 cells at dose of 400 μg/mL (Figure 1A). Further, we observed the cell morphology by scanning electron microscopy (SEM). As seen in Figure 1B, M0 and M2 macrophages had a spherical shape and an uneven surface. M1 macrophages were flat and had tentacles that protruded from the cell surface, indicating that M1 macrophages have superior phagocytic capability. MSGA-incubated macrophages were more inclined to the morphology of M1 phenotype.

To verify that MSGA polarized macrophages to M1 phenotype, we labeled M1/M2 macrophages with the surface markers CD86 and CD206, respectively. Flow cytometry analysis was used to analyze the phenotypic transformation of macrophages, and the gating strategy was shown in Supplementary Figure S1. Figure 1C revealed that MSGA significantly increased the proportion of CD86^+^ Raw264.7 cells from 0.88% to 4.03% (*p* < 0.001), while obviously decreased the percentage of CD206^+^ Raw264.7 cells from 58.1% to 32.4% (*p* < 0.001) at the dose of 400 μg/mL. Further evidence was demonstrated in BMDMs. Firstly, we successfully induced BNDMs (Supplementary Figure S1). MSGA increased the proportion of CD86^+^ BMDMs obviously (*p* < 0.001), and markedly decreased the percentage of CD206^+^ BMDMs (*p* < 0.001) at the dose of 100 μg/mL (Figure 1D). The results of immunofluorescence revealed that MSGA elevated the level of CD86 and negatively regulated the expression of CD206 in Raw264.7 cells (Figures 1E,F). According to these results, MSGA was able to polarize macrophages to CD86^+^ M1 phenotype.

### 3.2 MSGA increases the releases of inflammatory cytokines

**FIGURE 2:**
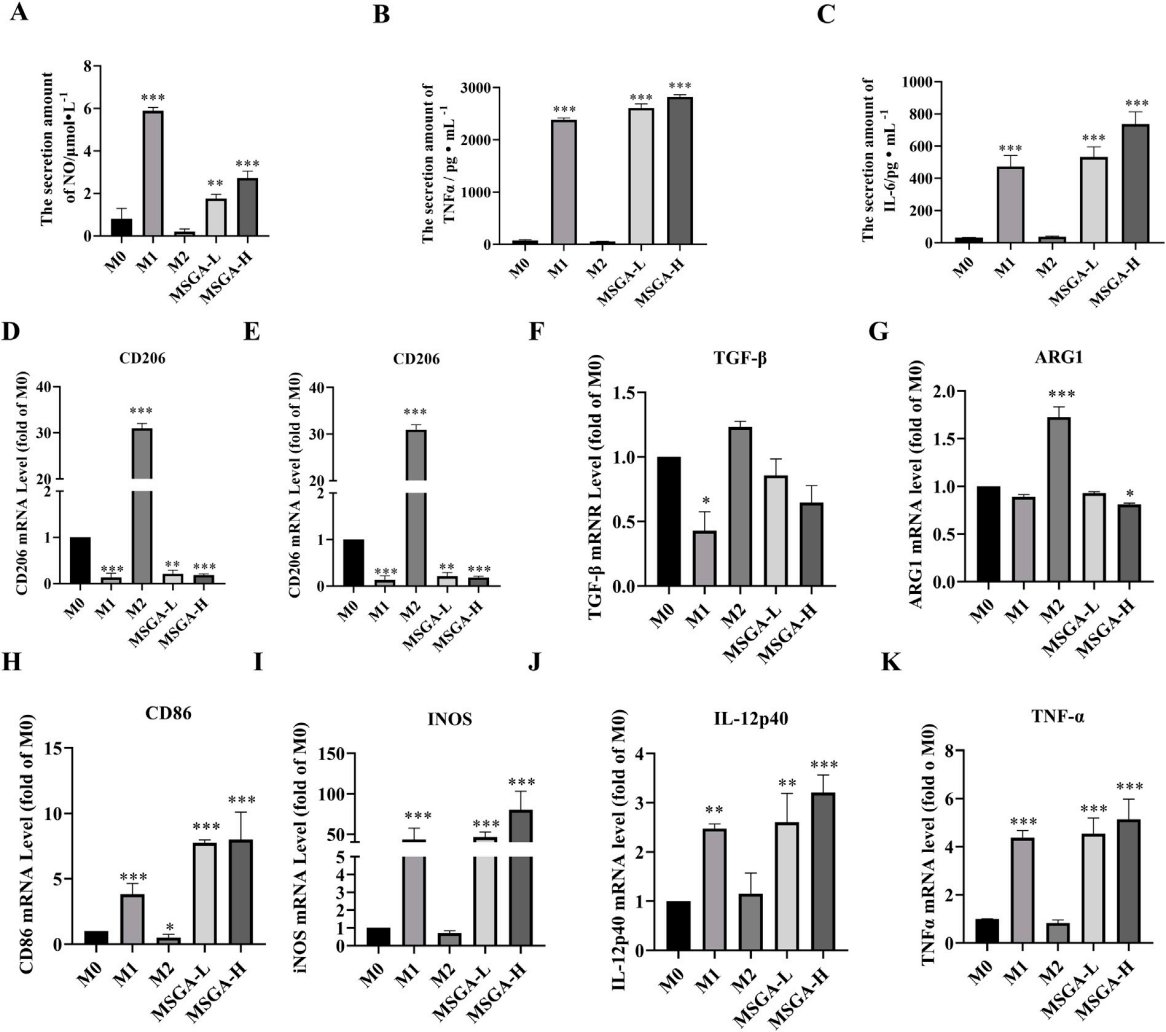
MSGA increases the releases of inflammatory cytokines. The contents of NO **(A)**, IL-6 **(C)** and TNFα **(B)** produced by MSGA-treated Raw264.7 cells were measured by ELISA, n = 6. The expressions of M1/M2 cytokines and surface markers in MSGA-treated macrophages were detected by real-time fluorescence quantitative PCR and expressed as a fold change compared with M0 macrophages, M2 biomarkers include CD206 **(D)**, IL-10 **(E)**, TGF-β **(F)** and ARG1 **(G)**. M1 biomarkers include CD86 **(H)**, INOS **(I)**, IL-12 **(J)** and TNFα **(K)**, n = 6. MSGA-L (100 μg/mL); MSGA-H (400 μg/mL). Data were expressed as means ± SD. One-way ANOVA with Tukey’s post-hoc test was used for the statistical analyses. Compared with M0 macrophages, statistically significant differences are indicated. ^*^ *p* < 0.05, ^**^ *p* < 0.01 and ^***^ *p* < 0.001.

M1 and M2 macrophages secrete different cytokines and mediate the anti-tumor/pro-tumor immune response of macrophages (Malekghasemi et al., 2020). We first determine the amounts of NO, IL-6 and TNFα in the cell supernatant. MSGA significantly promoted the contents of NO (*p* < 0.001), IL-6 (*p* < 0.001) and TNFα (*p* < 0.001) at the dose of 400 μg/mL (Figures 2A–C). Further, the relative mRNA expression levels of M1/M2 macrophage-associated cytokines were measured using qRT-PCR. As shown in Figures 2D–G, after incubation with Raw264.7 for 36 h, MSGA significantly decreased the expressions of CD206 (*p* < 0.001) and ARG1 (*p* < 0.05), which were identified as characteristic molecules of M2 macrophages at the dose of 400 μg/mL. Additionally, although MSGA down-regulated the expressions of M2 macrophage-related cytokines (IL-10 and TGFβ) at the mRNA level, but not significantly (Figures 2E,F). On the contrary, MSGA markedly rose the transcription levels of CD86 (*p* < 0.001) and M1 macrophage-specific cytokines (iNOS (*p* < 0.001), IL12-p40 (*p* < 0.001) and TNFα (*p* < 0.001)), especially in the high-dose group (Figures 2H–K).

### 3.3 MSGA reduces the cell viability of PDAC cells

**FIGURE 3:**
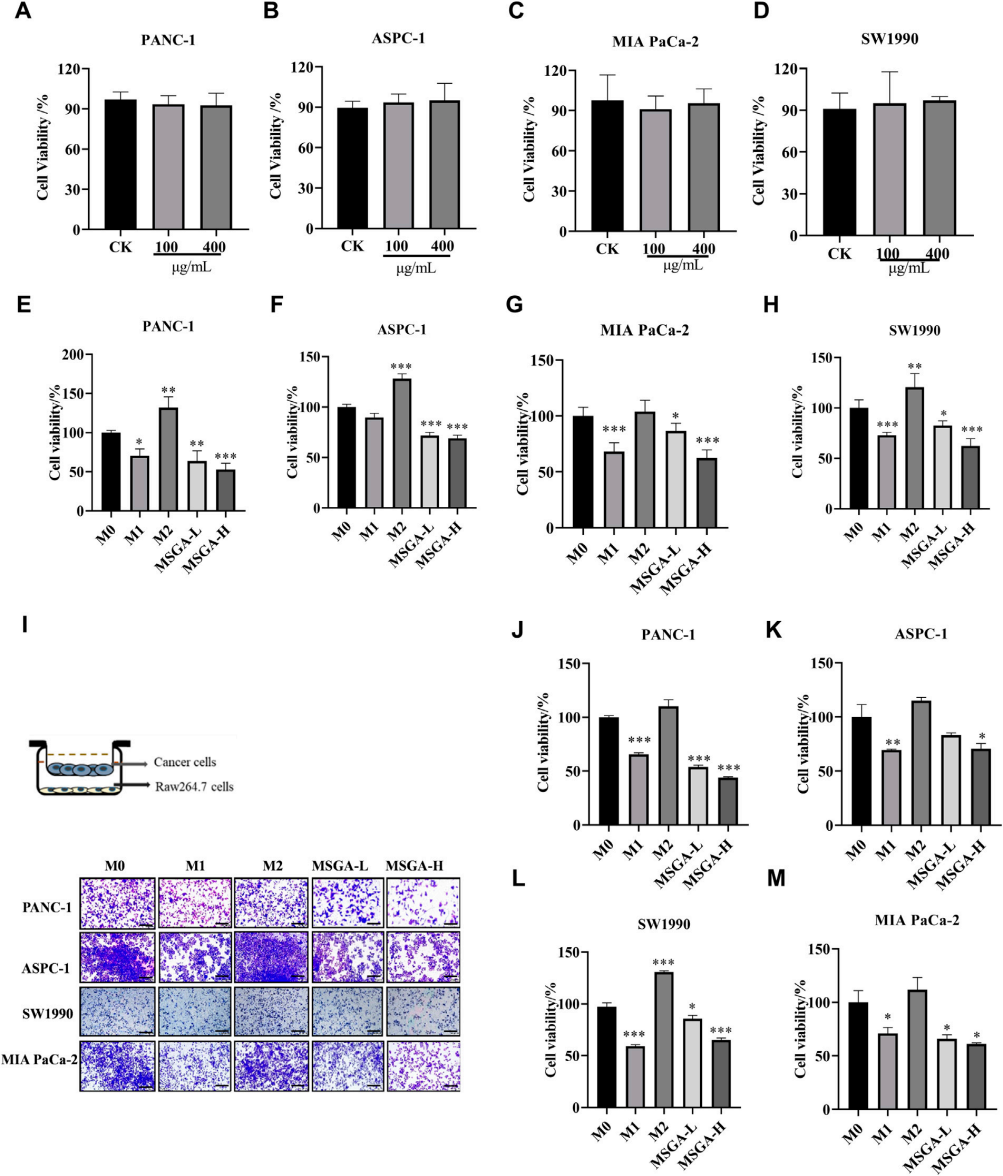
MSGA inhibits the viability of PDAC cells by polarizing macrophages into an M1 phenotype. Cytotoxicity of PANC-1 **(A)**, ASPC-1 **(B)**, MIA-PaCa-2 **(C)** and SW1990 **(D)** after MSGA treated cells for 36 h. Cytotoxicity of PANC-1 **(E)**, ASPC-1 **(F)**, MIA-PaCa-2 **(G)** and SW1990 **(H)** after different CMs treated cancer cells for 36 h. **(I)**. Different phenotypes of macrophages and MSGA pretreated macrophages were co-cultured with PDAC cells in transwell co-culture system. The representative images of the cancer cells were collected (scale bar, 100 μm), n = 3. The density of PANC-1 **(J)**, ASPC-1 **(K)**, SW1990 **(L)** and MIA-PaCa-2 **(M)** in upper chamber of transwell was analyzed by using Image **(J)**. MSGA-L (100 μg/mL); MSGA-H (400 μg/mL). One-way ANOVA with Tukey’s *post hoc* test was used for the statistical analyses. Compared with M0 macrophages, statistically significant differences are indicated. ^*^ *p* < 0.05, ^**^ *p* < 0.01 and ^***^ *p* < 0.001.

To investigate the anticancer activity of MSGA was mediated by M1 macrophages, we collected CMs of different treatment groups and constructed a transwell co-culture system. As shown in Figures 3A–D, MSGA had no direct cytotoxicities to PANC-1, ASPC-1, SW1990 and MIA-PaCa-2 cells. MSGA-CM significantly reduced the cell viabilities of PANC-1 (MASG-L, *p* < 0.01; MASG-H, *p* < 0.001), ASPC-1 (*p* < 0.001), SW1990 (MASG-L, *p* < 0.05; MASG-H, *p* < 0.001) and MIA-PaCa-2 (*p* < 0.001) in a dose-dependent manner (Figures 3E–H).

Furthermore, we simulated the coexistence environment of tumor cells and macrophages through the transwell co-culture system, and the representative images were presented in Figure 3I. Figures 3J–M showed that MSGA-CM-H restricted the growth of PANC-1 (*p* < 0.001), ASPC-1 (*p* < 0.05), SW1990 (*p* < 0.001) and MIA-PaCa-2 cells (*p* < 0.05) obviously, suggesting that the tumoricidal efficacy of MSGA was accomplished by programming macrophages transformation to the M1 type.

### 3.4 MSGA enhances the anti-PDAC activity of gemcitabine

**FIGURE 4:**
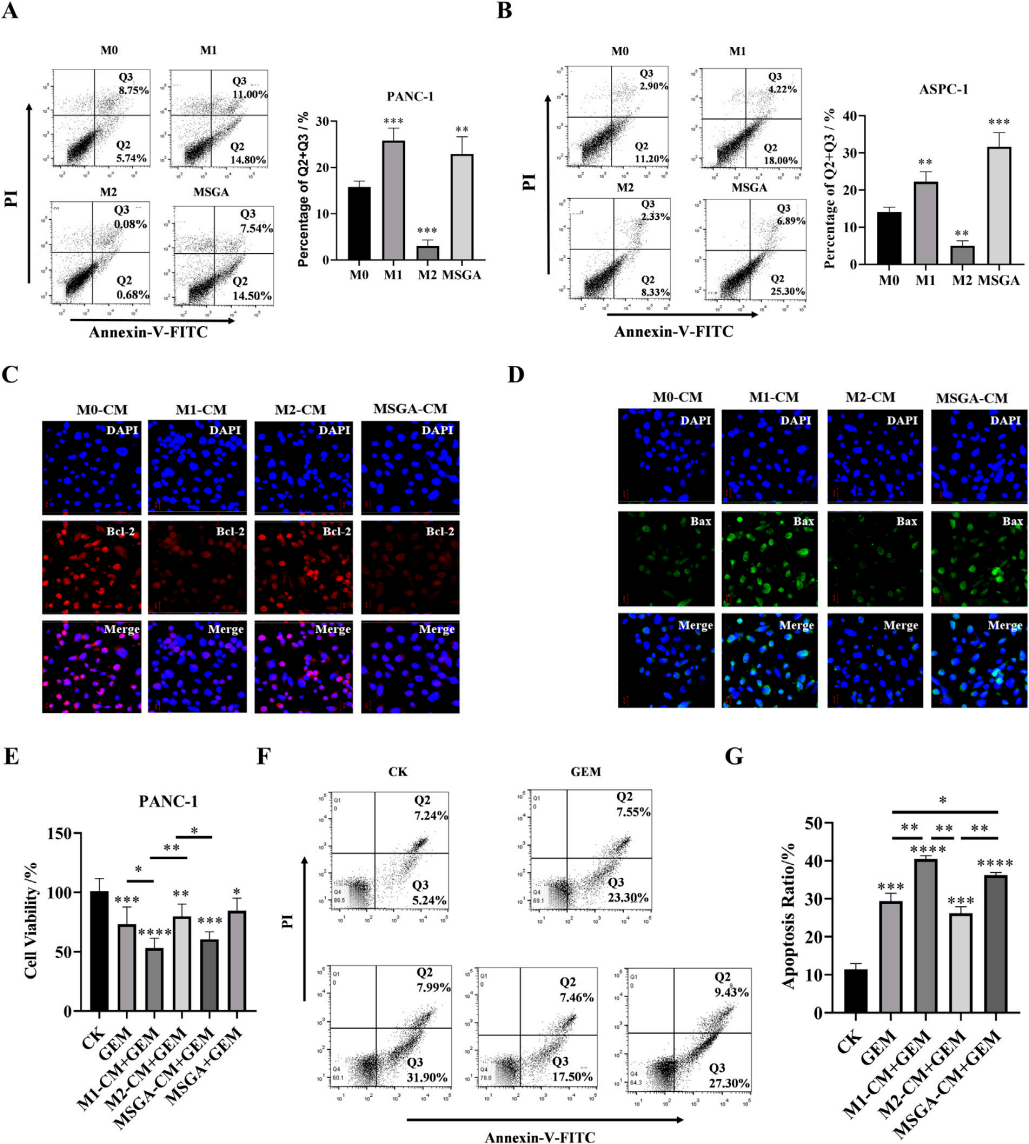
MSGA enhances the anti-PDAC activity of gemcitabine (GEM). The apoptosis rate of PANC-1 cells **(A)** and ASPC-1 cells **(B)** was detected after the cancer cells were incubated with different medium conditions (CMs) for 36 h, n = 3. Q1: necrotic cells, Q2, late apoptotic, Q3: early apoptotic, Q4: living cells. After PANC-1 cells were incubated with different CMs for 36 h, the expressions of Bcl-2 **(C)** and Bax **(D)** in cancer cells were labeled by immunofluorescence (IF), and representative images were collected by laser confocal microscopy (scale bar 20 μm, n = 3). **(E)**. Cytotoxicity of different conditioned mediums (CMs) and/or GEM on PANC-1 cells, n = 6. **(F)**. Apoptosis dection of PANC-1 cells after the cells treatment with GEM and/or different CMs for 36 h, n = 3. **(G)**. Statistical diagram of apoptosis rate of PANC-1 cells, n = 3. MSGA-L (100 μg/mL); MSGA-H (400 μg/mL). One-way ANOVA with Tukey’s *post hoc* test was used for the statistical analyses. Compared with M0 macrophages, statistically significant differences are indicated. ^*^ *p* < 0.05, ^**^ *p* < 0.01 and ^***^ *p* < 0.001.

M1 macrophage activation may make cancer cells more susceptible to apoptosis (Mills et al., 2016). Previous studies have shown that the a pyrazole derivative of curcumin (HC) reduced the level of pro-apoptotic proteins in breast cancer cells and EAC cells by polarizing macrophages into M1 phenotypes (Mills, 2015). This suggested that some natural products - induced M1 macrophages have the potential to induce apoptosis. Consistent with the above findings, we found that LPS and IFN-γ induced M1 macrophages significantly promoted the apoptosis of PANC-1 (*p* < 0.01) and ASPC-1 cells (*p* < 0.001) (Figures 4A,B). Consistent with the effect of M1 macrophages on PDAC cells, MSGA-CM obviously rose the apoptosis rate of PANC-1 cells (*p* < 0.01) and ASPC-1 cells (*p* < 0.001). Furthermore, we characterized the expression levels of apoptosis related protein (Bcl-2 and Bax) in macrophages. As seen in Figures 4C,D, MSGA-CM and M1-CM promoted the expression of Bax and suppressed the expression of anti-apoptotic protein Bcl-2 in PANC-1 cells. These results verified that MSGA-induced M1 macrophages could make cancer cells more susceptible to apoptosis.

To explore the effect of MSGA-polarized M1 macrophages on the anti-PDAC activity of gemcitabine (GEM), we treated PANC-1 cells with CMs of different treatment groups and/or 100 nM GEM for 24 h. The results showed that the chemotherapy drugs GEM, MSGA-CM + GEM and M1-CM + GEM both significantly reduced the cell viability of PANC-1 cells. Compared to M2-CM + GEM group, MSGA-CM + GEM and M1-CM + GEM also markedly increased the cytotoxicity of PANC-1 cells (*p* < 0.01 and *p* < 0.05) (Figure 4E). Apoptosis detection revealed that compared with GEM group, M1-CM + GEM (*p* < 0.01) and MSGA-CM + GEM (*p* < 0.05) both significantly raised the apoptotic rate of PANC-1 cells. (Figures 4F,G). The results exhibited that GEM’s pro-apoptotic activity was enhanced by MSCA-polarized M1 macrophages.

### 3.5 MSGA regulates JAK-STAT signaling pathway to polarize macrophages into M1 phenotype

**FIGURE 5:**
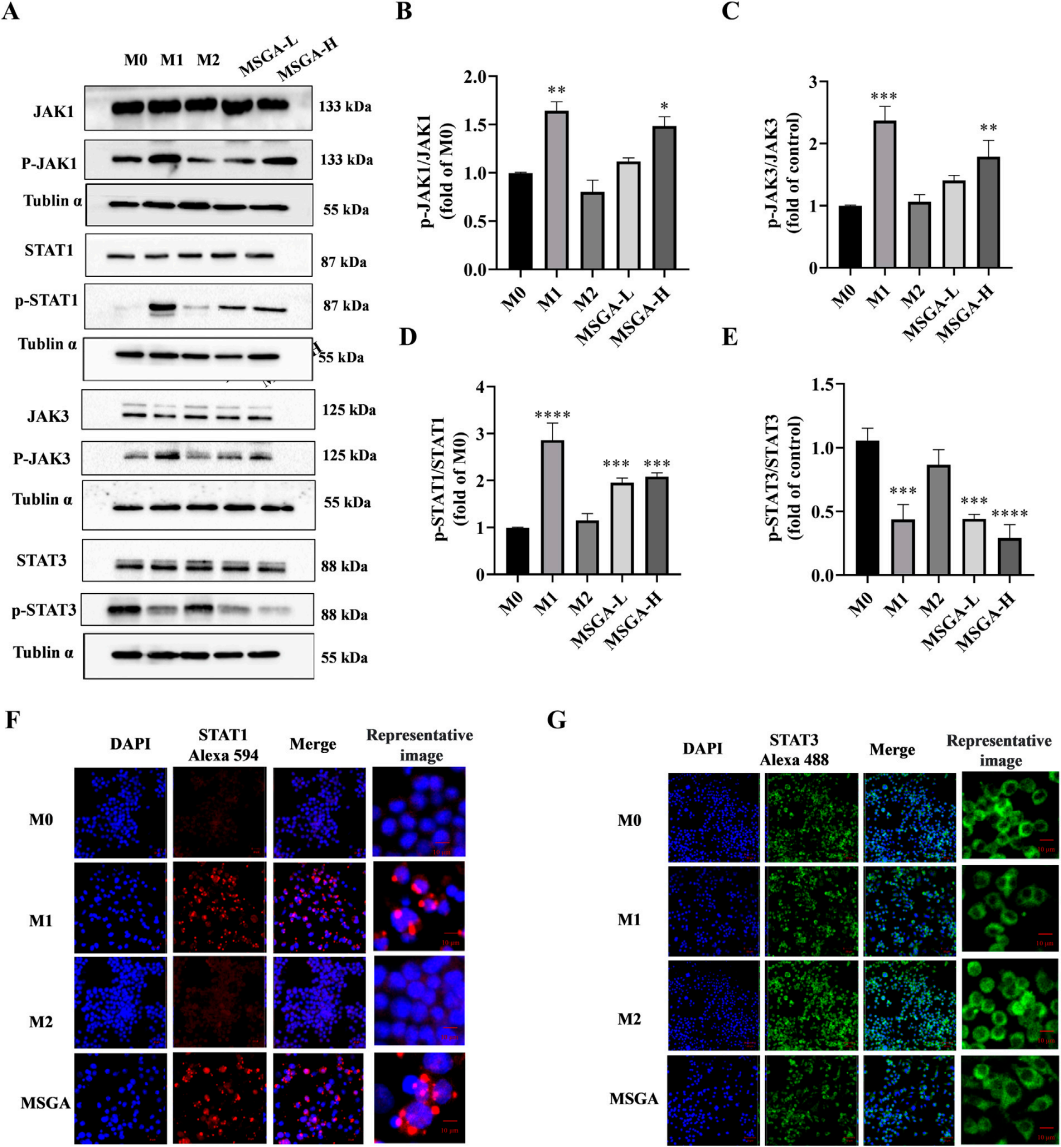
MSGA regulates JAK-STAT signaling pathway to polarize macrophages into M1 phenotype. **(A)**. The Western blot bands of MSGA regulates JAK-STAT signaling pathway, n = 3. **(B-E)**. Grayscale analysis of p-JAK1/JAK1, p-JAK3/JAK3, p-STAT1/STAT1, and p-STAT3/STAT3 expression. Immunofluorescence (IF) images of STAT1 **(F)** and STAT3 **(G)** were photoed by laser scanning confocal microscope (Scale bar, 20 μm; Scale bar in representative images, 100 nm), n = 3. MSGA-L (100 μg/mL); MSGA-H (400 μg/mL). One-way ANOVA with Tukey’s *post hoc* test was used for the statistical analyses. Compared with M0 macrophages, statistically significant differences are indicated. *p < 0.05, **p < 0.01 and ***p < 0.001.

The JAK-STAT pathway regulates a variety of biological activities in the body and mediates extensive immune responses, not only immune defense, but also processes that promote tumor cell survival, immune escape, and persistent inflammation (Hibbs et al., 1987). Recently, a growing amount of studies indicated that STATs plays a critical role in the modulation of macrophage polarization. STAT1 has been reported as an activator of M1 macrophage polarization (Yu et al., 2019). Consistent with previous reports, the obvious upregulation of phosphorylation of JAK1 (*p* < 0.01), JAK3 (*p* < 0.01) and STAT1 (*p* < 0.001) was observed in M1 macrophages. Treatment with MSGA significantly enhances the phosphorylation of JAK1 (*p* < 0.05), JAK3 (*p* < 0.01), and STAT1 (*p* < 0.001) in macrophages (Figures 5A–D). In most studies, activation of STAT3 stimulates macrophage M2 polarization (Ino et al., 2013; Yang and Zhang, 2017). In our results, the phosphorylation of STAT3 was obviously downregulated in M1 macrophages (*p* < 0.001) and MGSA-treated macrophages (*p* < 0.001) (Figures 5A,E). Furthermore, the expression of STAT1/3 in macrophages was visually characterized by immunofluorescence. As displayed in Figures 5F,G, MSGA and M1 macrophages promoted the translocation of STAT1 into the nucleus. Conversely, we observed that STAT3 was excluded from the periphery of the nucleus indicating that STAT3 phosphorylation was inhibited in the MSGA group. These further explained that MSGA transformed macrophages to the M1 phenotype by activating JAK1/3-STAT1 and inhibiting STAT3 nuclear translocation.

## 4 Discussion

Glycogen is a long-term reservoir of glucose, produced mainly by liver and muscle cells. It is commonly accepted that glycogen offers cells energy and maintains blood glucose homeostasis. Despite this primitive knowledge, the physiological and pathological function of glycogen may be more multifaceted. MSGA is glycogen isolated from *Strongylocentyotus internedius*, and prior research has indicated that MSGA showed pro-inflammatory activity. In this work, we concentrated on the role of MSGA in the phenotypic remodeling of macrophages and its anti-PDAC effect when employed as an immune stimulant in conjunction with gemcitabine.

In addition to energy storage, various physiological functions of glycogen have been reported. Glycogen metabolism regulates tumor growth. Study have shown that glycogen synthesis was enhanced in early tumors, and restriction of glycogenolysis hindered the production of new fatty acids, nucleic acids and glucose, thus reducing the growth of cancer cells and making them more susceptible to apoptosis (Kumari et al., 2019). Glycogen metabolism is involved in dendritic cell (DC) maturation and macrophage activation.Toll-like receptor (TLR) and Syk-Dependent C-type lectin receptor (CLR) agonists could support early recombination of glycolysis and stimulation in DCs by promoting glycogen metabolism. (Hu et al., 2021). Glycogens extracted from different species have been reported have anti-cancer effect or immunostimulatory activity, such as *Strongylocentrotus nudus, Cyclina sinensi,* scallop, bovine and honeybee larvae (Laskin et al., 2011; Mantovani et al., 2010; Gordon and Taylor, 2005; Lee et al., 2004). In this research, we studied the tumor immunomodulatory activity of a glycogen extracted from *Strongylocentyotus internedius*. MSGA drived macrophages differentiation to the M1 subtype, which secretes pro-inflammatory cytokines (e.g., iNOS, TNFα) to suppress cancer cell viability. Studies reported by Kakutani revealed a correlation between glycogen’s molecular weight and its immunoregulatory action. Glycogens with molecular weights more than 1.0 × 10^7^ barely stimulated Raw264.7 cells, whereas glycogens of 5.0–6.5 × 10^6^ had strong activation effect on Raw264.7 cells (Favaro et al., 2012; Ma et al., 2020). Contrary to the above conclusion, the molecular weight of MSGA is 2.65 × 10^7^ Da, and it activated Raw264.7 cells strongly. Generally, glycogen has a branch every 12 residues and a molecular weight of millions to tens of millions Da (often less than 10^7^ Da) (Kumari et al., 2019). Compared to previous reports, MSGA has a higher molecular weight and more dense branching. Some previous studies have also reported that glycogen with denser branch structures induces anti-tumor immune responses (Gong et al., 2020; Nagasaki et al., 2021). These findings indicated that the immunoregulatory effect of glycogen is not solely dependent on its molecular weight. We speculated that the high degree of branching may contribute to the immunostimulatory activity of glycogen.

Glycogen-induced immune activation involves sophisticated regulatory mechanisms. A glycogen (SEP) extracted from *S. nudus* inhibited tumor growth in mice by up-regulating the phosphorylation and transcription levels of ERK in spleen, promoting splenocyte proliferation and increasing CD4^+^ and CD8^+^ T cell numbers (Mantovani et al., 2010). Kakutani et al. reported that enzymatic synthesis of glycogen (ESG) directly interacted to TLR2 and enhanced the activity of NFκB and MAPK, thus facilitating the various innate immune responses of macrophages (Vinogradov et al., 2014; Huang et al., 2021). This suggested that immune activation is a means for glycogen to resist tumors. Currently, there is a lack of evidence regarding glycogen’s regulatory effects on the JAK-STAT signaling pathway. In this study, we found that MSGA modulate the JAK-STAT signaling pathway. In response to different signaling molecules, the JAK-STAT pathway regulates proliferation, activation, homeostasis, and function of immune cells. IFNγ-mediated JAK-STAT signalling pathway plays an essential role in M1/M2 phenotypic transition of macrophages (Hibbs et al., 1987; Yang and Zhang, 2017). Phosphorylated STAT1 can be relocated to the nucleus, and then type I IFNs and M1-related genes, such as NOS2, IL-12, IL-2, and MHCII are activated and begin to transcription (Yu et al., 2019). Conversely, activated STAT3 encourages M2 differentiation and elicits the generation of M2-related cytokines (Ino et al., 2013; Yang and Zhang, 2017; Besford et al., 2020). We found that MSGA exhibited differential regulatory activity towards STATs. MSGA activated the JAK1/3-STAT1 signaling pathway but downregulated STAT3 phosphorylation, promoting macrophage polarization to the M1 phenotype. Our previous studies have found that MSGA upregulated the phosphorylation levels of NFκB and MAPK to activate macrophages. MDSCs activated the transcription factors STAT1 and NF-κB, which translocate to the nucleus. Their crosstalk may regulate transcriptional synergy via the coactivator CBP (CREB binding protein) (Chen et al., 2020). Interestingly, in addition to regulating the immune response, activation of STAT1 increased the apoptosis of cancer cell and suppressed tumor cell proliferation. In contrast to STAT1, STAT3 is a downstream oncogenic mediator that controls cell cycle progression and cell apoptosis (Sun et al., 2020; Su et al., 2019). In this study, MSGA did not directly affect the cell viabilities of PDAC cells. Collectively, JAK-STAT signal pathway was involved in the MSGA-induced M1 phenotypic transformation of macrophages, and MSGA affected the survival and promoted apoptosis of cancer cells by shifting macrophages to the M1 phenotype.

Chemotherapy resistance is one of the key elements influencing the effectiveness of gemcitabine, resulting in poor prognosis of patients (Chen et al., 2021). TAMs affect the uptake of gemcitabine by PDAC. In addition, treatment with gemcitabine causes a change in innate immune cells, including more protumoral M2 macrophage infiltration and metabolic reprogramming. Macrophages programmed by PDAC cells release a range of pyrimidine species and metabolic enzymes, including deoxycytidine and cytidine deaminase, which inhibit gemcitabine and induce chemoresistance through molecular competition at the level of drug uptake and metabolism (Kakutani et al., 2007; Kakutani et al., 2008; Yan et al., 2011). The above studies supported that depleting TAMs or reprogramming macrophages improved chemotherapy resistance and enhances chemosensitivity to gemcitabine. Studies have demonstrated that depleting TAMs or reprogramming macrophages with appropriate components in combination with gemcitabine enhanced antitumor activity and alleviated chemotherapy resistance of gemcitabine (Guo et al., 2009; Lawrence and Natoli, 2011; Peng et al., 2020). We found that MSGA promoted apoptosis of PDAC cells by programming macrophages to the M1 phenotype. When combined with gemcitabine, MSGA enhanced the anti-PDAC activity of gemcitabine by enhancing the pro-apoptotic ability. Collectively, the combination of MSGA and gemcitabine may provide an alternative treatment for PDAC.

The bioavailability of polysaccharides is a key obstacle to their clinical application. Although studies have shown that large-molecular-weight glycogen could exert immune-stimulating activity in mice, there is still a lack of relevant research on their absorption, distribution and metabolism patterns in the body (Gong et al., 2020; Wang et al., 2014). Our research lacks studies on the *in vivo* immune activity of Msga, which is also a key limitation of this study.

Our research revealed that MSGA activated JAK1/3-STAT1 and downregulated STAT3 phosphorylation to reprogram macrophages into an anti-tumor M1 phenotype. MSGA-programmed M1 macrophages increased the anti-tumor activity of GEM by promoting apoptosis of cancer cells. Further evaluation of tumor immune activity *in vivo* will be studied in the future. This study provides a basis for the application of MSGA in the immunotherapy of PDAC.

## Data Availability

The raw data supporting the conclusions of this article will be made available by the authors, without undue reservation.
