# Supplementary material for: A glycogen derived from sea urchin-Strongylocentyotus internedius shifts macrophages to the M1 phenotype and enhances the anti-pancreatic cancer activity of gemcitabine
Source: Front Pharmacol. 2025 Jul 25;16:1600349. doi: 10.3389/fphar.2025.1600349 (PMC12331687; doi:10.3389/fphar.2025.1600349)
Supplement: Supplementary file 1 [file DataSheet1.docx]

Supplementary Material

## Supplementary Figures

**
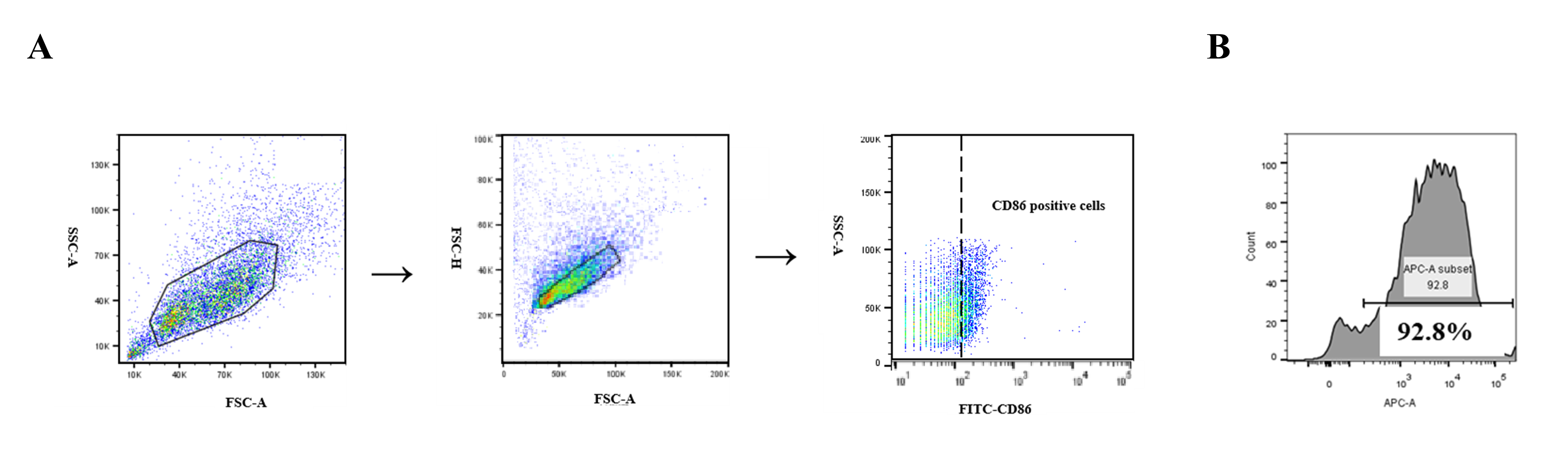
**

**Supplementary Figure 1.** Gating strategies for flow analysis of macrophage polarization.

**
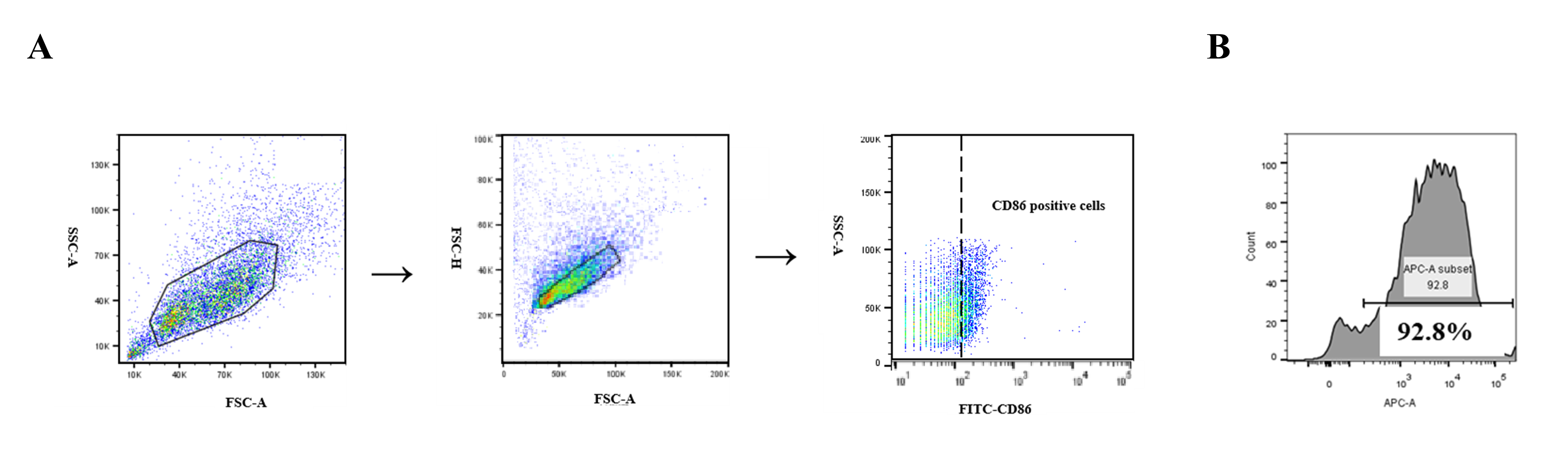
**

**Supplementary Figure 2.** The ratio of bone marrow derived macrophages (BMDMs).
